# Supplementary material for: Dual parallel stream-specific and generalized effects of corticogeniculate feedback on LGN neurons in primate and carnivore
Source: Nat Commun. 2025 Apr 9;16:3380. doi: 10.1038/s41467-025-58667-9 (PMC11982367; doi:10.1038/s41467-025-58667-9)
Supplement: Supplementary file 1 — Supplementary Information [file 41467_2025_58667_MOESM1_ESM.pdf]

Dual parallel stream-specific and generalized effects of corticogeniculate feedback on LGN neurons in primate and carnivore

Supplemental Figures and Tables

Supplemental Figure 1

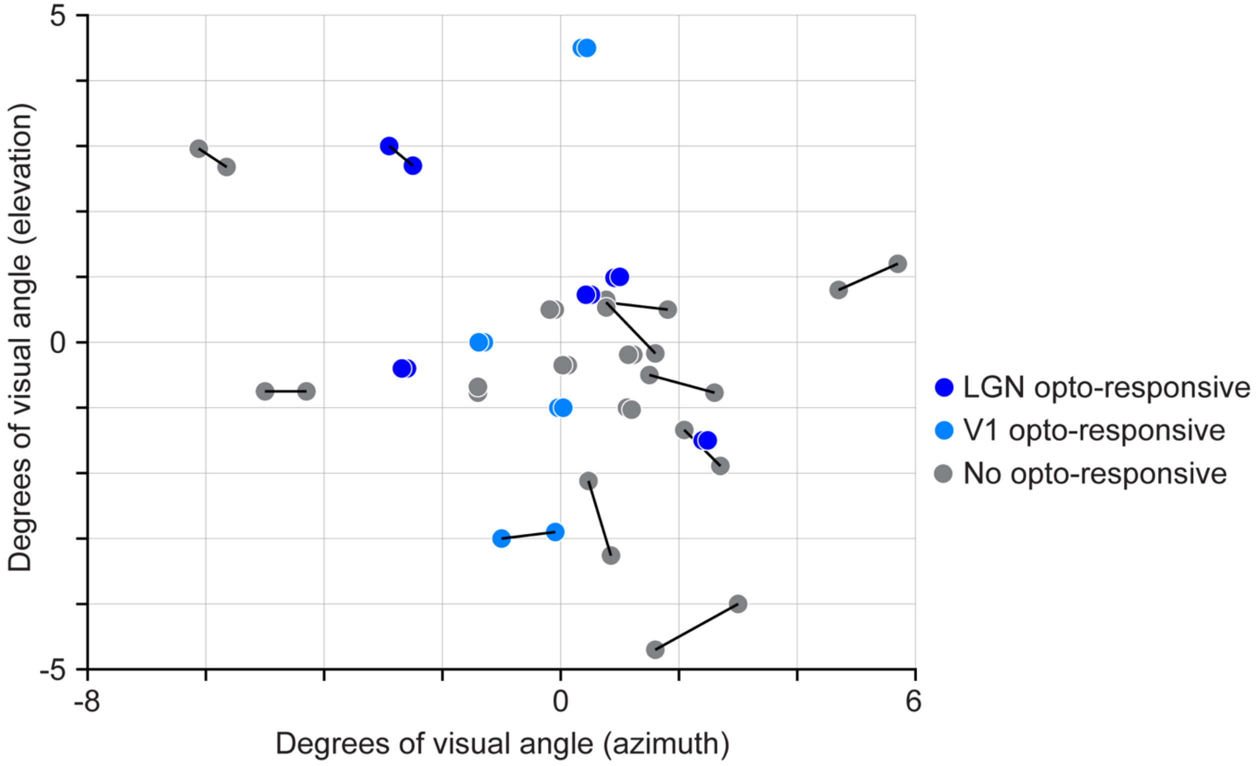

Supplemental Figure 1: Receptive field overlap for simultaneously recorded LGN and V1 neurons. Pairs of dots represent centers of receptive fields of simultaneously recorded LGN and V1 neurons. Pairs with minimal or zero distance between receptive field centers are represented by adjacent dots, otherwise black lines indicate distance between receptive field centers per pairing. Dark blue dots represent pairs in which one or more LGN neurons were directly modulated by the LED alone, light blue dots represent pairs in which one or more V1 neurons were LED-modulated, grey dots represent pairs in which no neurons were directly modulated by the LED alone. Note: plot origin is the center of the monitor, not foveal center, as precise eye position was not known. LGN and V1 recordings were all made from parafoveal eccentricities based on electrode positions within LGN and opercular V1.

Supplemental Figure 2

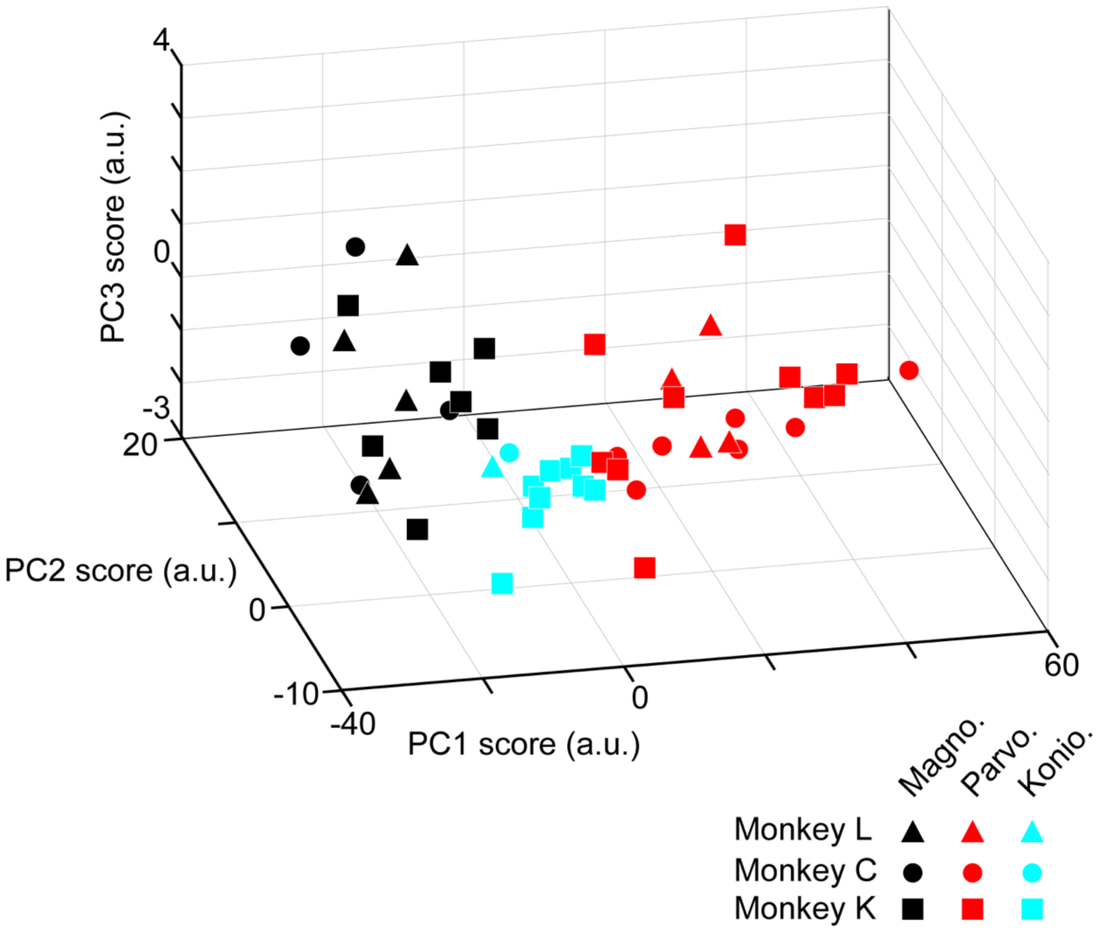

Supplemental Figure 2: Clustering of LGN neurons based on tuning metrics. Principal components analysis scores for the first 3 principal components (PC1, 2, 3) from analysis of tuning metrics: c50, preferred temporal frequency, preferred spatial frequency, and surround suppression index for 48 LGN neurons, color coded according to qualitative assignments as parvocellular (red), magnocellular (black), or koniocellular (blue) LGN neurons. Symbols indicate from which animal data were obtained (triangles for Monkey L, circles for Monkey C, squares for Monkey K). Note: LGN neurons of the same type recorded in different monkeys showed remarkable similarity in their multi-dimensional tuning properties (different symbols of the same color are grouped).

Supplemental Figure 3

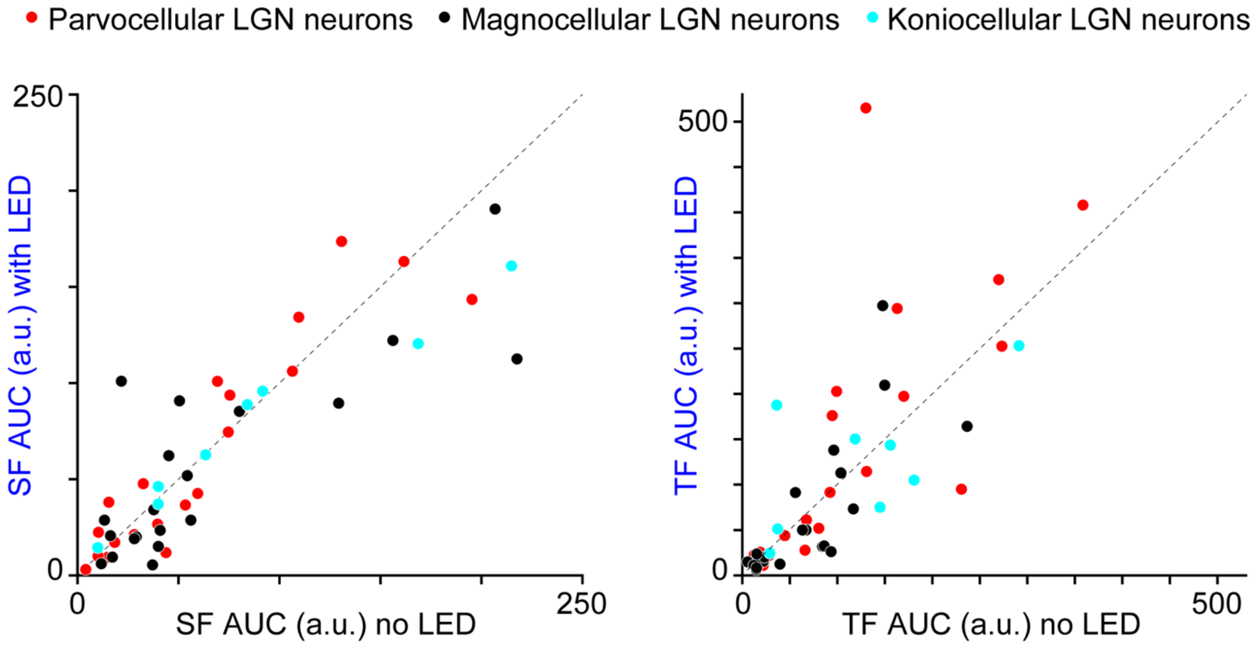

Supplemental Figure 3: Gain measures are not different across LED conditions. Gain values (area under the curve, AUC) for parvocellular (red), magnocellular (black), and koniocellular (cyan) LGN neurons across conditions without (x-axis) and with LED stimulation (y-axis, blue label). There are no differences in AUC values computed from spatial frequency (SF) curves (left) or temporal frequency (TF) curves (right) across LED conditions. See **Table 2** for all statistics.

Supplemental Figure 4

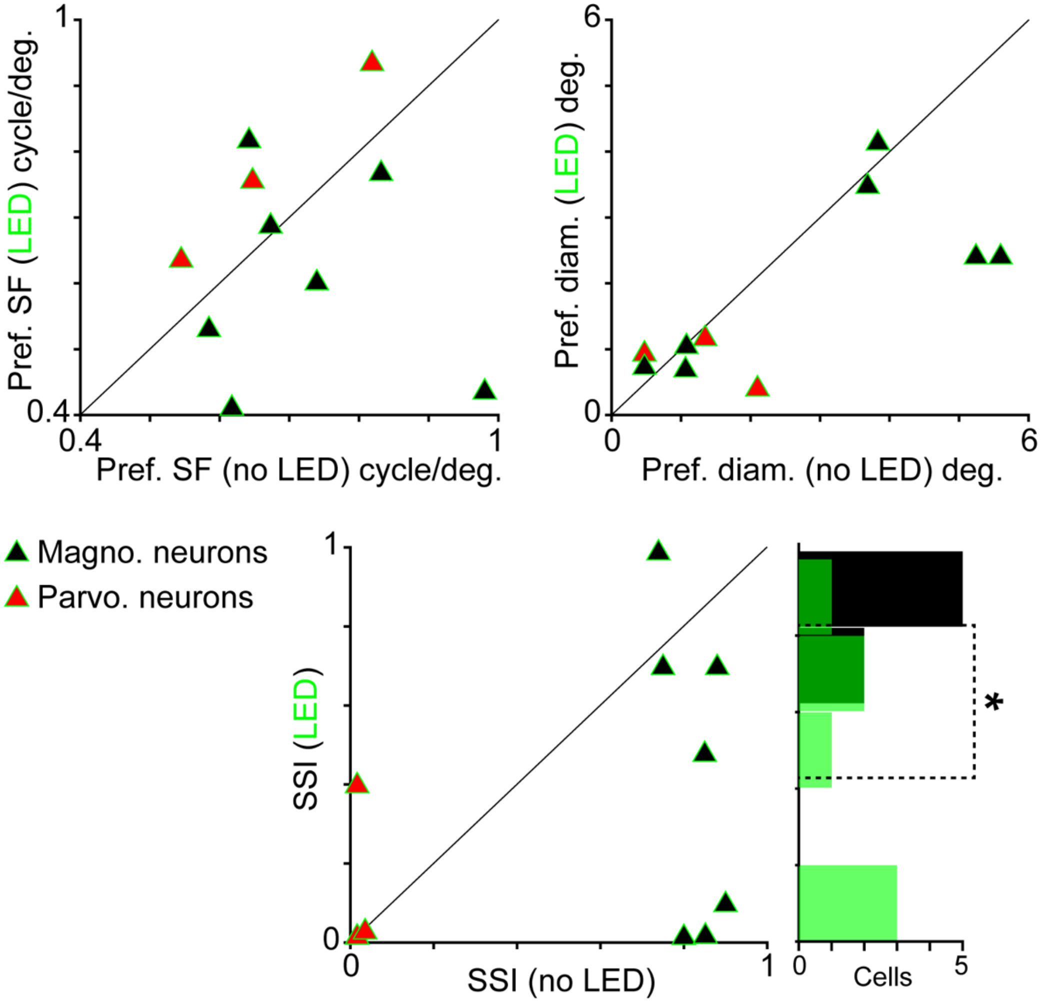

Supplemental Figure 4: Tuning metrics across ArchT-stimulating LED conditions. Tuning metrics for parvocellular (red fill, green outline) and magnocellular (black fill, green outline) LGN neurons (all from Monkey L) across conditions without and with Arch T-LED stimulation of CG feedback showing preferred spatial frequency (SF, top left), preferred grating diameter, and surround suppression index (SSI, bottom right). Distribution right of the bottom plot illustrates difference in SSIs across LED conditions for sampled magnocellular neurons only, dashed line indicates averages per condition, asterisk indicates significant difference (p=0.049, paired t-test; average SSI for 7 magnocellular LGN neurons without LED=0.82±0.03, average SSI with LED=0.43±0.16). There were no differences in preferred spatial frequency or grating diameter across LED conditions (p>0.21 for both).

Supplemental Figure 5

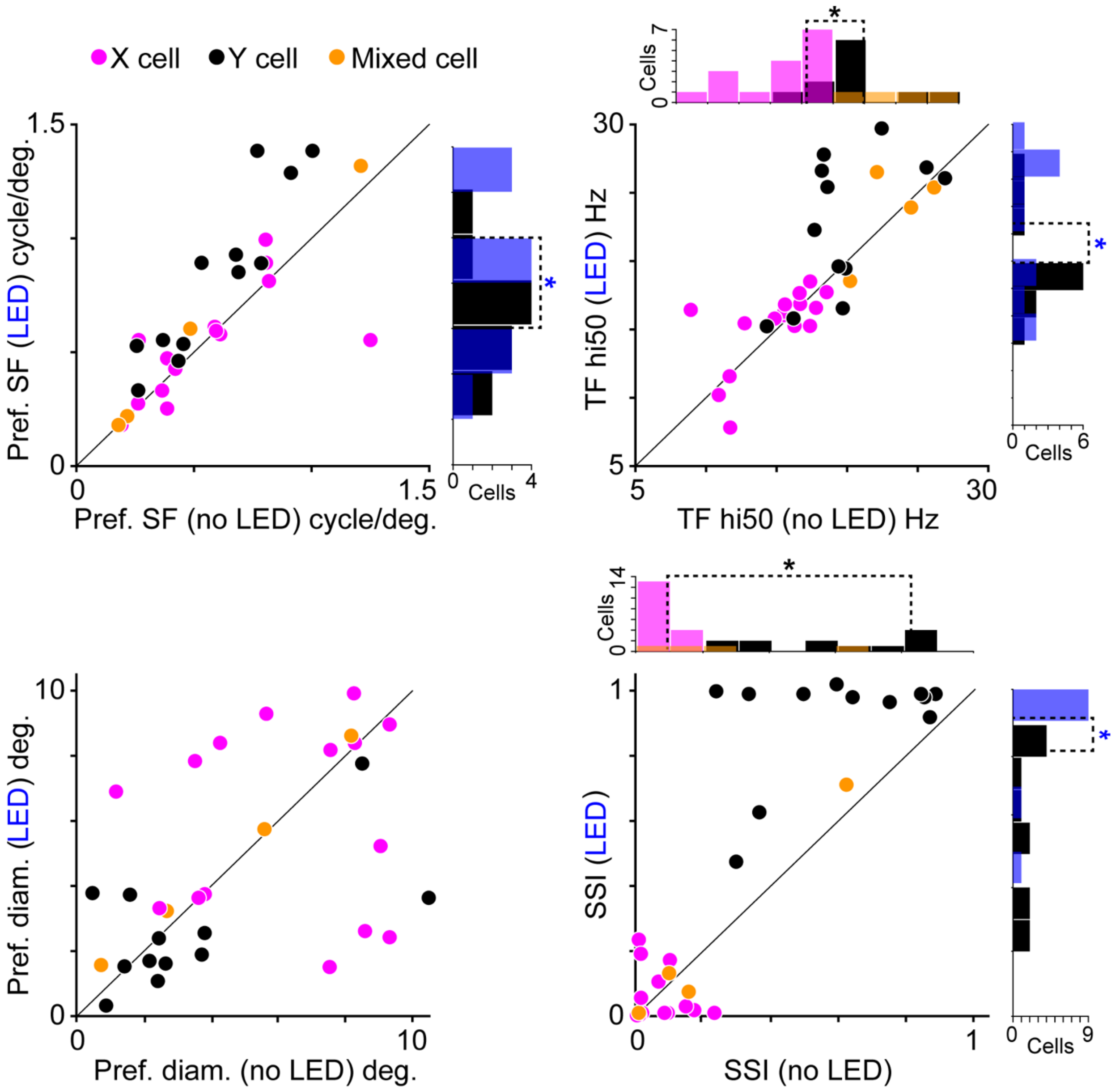

Supplemental Figure 5: Tuning metrics differ between LGN neuronal types and across LED conditions in ferrets. Tuning metrics for all sampled X (magenta), Y (black), and mixed (orange) LGN neurons across conditions without and with LED stimulation of CG feedback: preferred spatial frequency (SF, top left), temporal frequency high50 (TFhi50, top right), preferred grating diameter (bottom left), and surround suppression index (SSI, bottom right). Distributions above rightmost plots illustrate differences between sample X and Y neurons, dashed lines indicate averages per cell type, black asterisks indicate significant differences (see **Supplemental Table 1** for statistics). Distributions to right of plots illustrate differences across LED conditions for sample Y neurons only, dashed lines indicate averages per condition, blue asterisks indicate significant differences (see **Supplemental Table 1** for statistics).

Supplemental Table 1: Tuning metrics for LGN neurons in ferrets. Average contrast to evoke a half-maximal response (c50), preferred spatial frequency (SF), preferred temporal frequency (TF), temporal frequency to evoke a half-maximal response greater than the preferred frequency (TF high50), preferred grating size, and surround suppression index (SSI) for X and Y LGN neurons; for each metric, average values without LED at left, with LED at right (blue shading; LED labels only shown for c50 columns). Bold blue p-values indicate significant effects of the LED (independent paired t-tests), using a Bonferroni corrected alpha=0.0125 for contrast, SF and TF metrics and a Bonferroni corrected alpha=0.025 for size metrics. Bold black p-values indicate significant differences across neuronal types (comparisons of no LED responses using two-sided Wilcoxon signed-rank tests).

	**c50 No LED / LED**		**Preferred SF**		**Preferred TF**		**TF high50**		**Preferred grating size (deg.)**		**SSI**	
X neurons (n=22)	52.9±3.5	51.9±4	0.5±0.07	0.5±0.07	5.9±1.3	5.9±1.3	16.6±1	16.9±1	5.8±0.6	6±0.6	0.09±0.03	0.1±0.04
LED effect: P values (paired t-test)	0.73		0.99		0.97		0.63		0.78		0.85	
Y neurons (n=12)	22.7±4	26±4.2	0.6±0.8	0.8±0.1	12.6±2.1	14.9±2.3	19.8±1.1	22.6±1.5	3.4±0.9	2.7±0.6	0.85±0.25	0.91±0.05
LED effect: P values (paired t-test)	0.42		**0.0005**		0.22		0.04		0.33		**0.0007**	
X vs Y differences (unpaired t-test)	**4.8×10^{-6}**		0.33		**0.009**		**0.03**		**0.04**		**8.2×10^{-6}**	
